# Genome Sequences of *Gordonia rubripertincta* Bacteriophages MajinBuu and Coldpizza

**DOI:** 10.17912/micropub.biology.002379

**Published:** 2026-10-29

**Authors:** Sarah E Beatty, Maritza Cervantes, Hope M Dunn, Lissette E Garcia, Gerlyn M Fortuna, Esmond C Fung, Maddisson S Harmon Petroff, Itzayana B Parker, Kendra W Kimberley, Chelsey C McKenna, James R Theoret, Erin J Windsor, Earl J Yoon

**Affiliations:** 1 Department of Biological Sciences, College of Southern Nevada, Las Vegas, NV, United States

## Abstract

MajinBuu and Coldpizza are bacteriophages isolated on *Gordonia rubripertincta* NRRL B-16540. MajinBuu has a 61,525 bp genome with a 3' overhang and 92 predicted genes, while Coldpizza's 61,651 bp circularly permuted genome encodes 79 predicted genes. Neither genome is predicted to contain genes encoding tRNAs or tmRNAs.

**Table 1. Genomic Characteristics of Bacteriophages MajinBuu and Coldpizza:** Information about the discovery, genome characteristics, and sequencing for MajinBuu and Coldpizza.

**Table:** 

Phage	MajinBuu	Coldpizza
GPS Coordinates	36.28765 N, 115.31643 W	40.440107 N, 79.958725 W
Sequencing Coverage	3,210	1,406
Number of Reads	2,015,125	1,406
Genomic Length (bp)	61525	61651
GC Content (%)	51.60%	67.60%
Genome End Type	3’ Single-stranded Overhang (nine bases)	Circularly Permuted
Putative Genes	92	79
Cluster	DJ	DR

## Description

Bacteriophages are diverse and abundant in terrestrial systems, and it is estimated that there are 10 ^9^ phages per gram of soil (Hatfull, 2015). MajinBuu and Coldpizza are two bacteriophages that infect the host bacterium, *Gordonia rubripertincta* NRRL B-16540. They were isolated from soil and characterized through the Science Education Alliance-Phage Hunters Advancing Genomics and Evolutionary Science (SEA-PHAGES) program (Russell and Hatfull, 2017). *Gordonia* are bacteria commonly found in soil and associated with wastewater in treatment plants. They are also opportunistic pathogens that can cause catheter infections and other infections in immunocompromised patients (Pollenz et al., 2022).

MajinBuu and Coldpizza were isolated from soil samples collected in a park in Las Vegas (GPS coordinates: 36.28765 N, 115.31643 W) using a direct isolation method and from a plant bed in Pittsburgh (GPS coordinates: 40.440107 N, 79.958725 W) using an enriched isolation method, respectively (Zorawik et al., 2024). Briefly, the MajinBuu soil sample was incubated in nutrient broth with 2.5 μg/mL Amphotericin B and 4.5 mM CaCl _2,_ and the Coldpizza soil sample was incubated in PYCa (peptone-yeast-calcium) broth. Both suspensions were incubated at 30°C with shaking at 250 rpm for one hour. The suspensions were spun in a centrifuge at 2,000 x g for 10 minutes, and the supernatants were filtered through 0.22 μm filters. For direct isolation, the supernatant was used directly for plaque assays by plating in top agar with *Gordonia rubripertincta* NRRL B-16540. For enriched isolation, 10 mL of supernatant was inoculated with 500 μL of *G. rubripertincta* and incubated at 30°C for three days on a rocker at 220 rpm. The resulting culture was filtered through a 0.22 μm filter prior to the plaque assay. After completing three rounds of picking isolated plaques and plating to purify the phages, and preparing liquid lysates of these phages, DNA was extracted from the lysates using the Norgen phage DNA isolation kit, which included five rounds of freeze/thaw (4-minute freeze in a dry-ice methanol bath and a 1-minute thaw in 60°C water) **.** MajinBuu forms clear round plaques with defined borders that have an average diameter of 1.06 mm (plaque diameter based on measurements of five plaques from a single plate). Coldpizza forms smaller round clear plaques with defined borders that have an average diameter of 0.65 mm (plaque diameter based on measurements of five plaques from a single plate). Plaque measurement analysis for both phages was performed using ImageJ (Schneider et al., 2012).

Sequencing libraries were created from extracted genomic DNA using New England Biolabs Ultra II Library kit, before sequencing at the Pittsburgh Bacteriophage Institute using an Illumina NextSeq 1000 instrument (X-LEAP P1 kit). The resulting 100-base single-end reads were trimmed by cutadapt 4.7 (using option: –nextseq-trim 30) and skewer 0.2.2 (using options: -q 20 -Q 30 -n -l 50) and then assembled using Unicycler version 0.5.1 (Martin, 2011; Jiang et al., 2014; Wick et al., 2017). Assemblies were visualized using Consed v29 and checked for accuracy and completeness by looking for gaps and low consensus areas (Gordon et al., 1998). Bacteriophage genomic termini were determined via analysis of read start pileups and coverage as previously described (Russell, 2018). Sequencing and assembly results are presented in Table 1.

Annotations were completed using the following programs: DNA Master v2.53.6 (http://cobamide2.bio.pitt.edu/computer.htm), Starterator v1.2 ( https://github.com/SEA-PHAGES/starterator ), Phamerator v2.5 (Actino_Draft database, https://phamerator.org/ ) (Cresawn et al., 2011), PhagesDB BLAST (database: actinobacteriophage database; https://phagesdb.org ) (Russell and Hatfull, 2017), NCBI BLAST (Databases: blastn standard core nucleotide and blastp standard non-redundant) (Altschul et al., 1990), PECAAN (Rinehart et al., 2016), GeneMark v2.5p (Besemer and Borodovsky, 2005), Glimmer v3.02 (Delcher et al., 2007), Aragorn (v1.1 and v1.2.38) (Laslett and Canback, 2004), HHPRED (Databases: PDB, NCBI-CD, PFam-A, and SCOPe) (Gabler et al., 2020), tRNAscan-SE v2.0 (Lowe and Chan, 2016), DeepTMHMM v1.0.24 (Hallgren et al., 2022), all using the default settings.

MajinBuu has 92 predicted genes that are transcribed unidirectionally, and 26 of these genes were assigned putative functions. Coldpizza has 79 genes, and 31 were assigned putative functions. MajinBuu and Coldpizza were assigned to clusters DJ and DR, respectively, based on gene content similarity of at least 35% to the phages in the Actinobacteriophage database, phagesdb ( https://phageDB.org ) (Pope et al., 2017; Russell and Hatfull, 2017). As with other cluster DJ phages, genes associated with lysis in MajinBuu, including *lysA* , *lysB* , and those encoding holin, are flanked by a conserved set of genes encoding proteins of unknown function that contain transmembrane domains. The proximity and synteny of these transmembrane-containing proteins to lysis genes suggests involvement in lysis and may define a lysis cassette (Bendele et al., 2025). Unlike all cluster DR phages, where both *lysA* and *lysB* genes could be identified, no *lysB* could be identified for Coldpizza. Where Lysin A is typically essential for phage replication, Lysin B is not (Payne et al., 2009). Both genomes do not contain any predicted tRNA or tmRNA genes, which is typical for both clusters. Similarly, neither phage is predicted to be lysogenic due to the absence of identifiable immunity repressor or integrase genes.

**Data Availability.** MajinBuu GenBank and SRA accession numbers are PV915850 and SRR34858428 , respectively. Coldpizza GenBank and SRA accession numbers are PV915803 and SRR34858419 , respectively.
